# Yak meat content in feed and its impact on the growth of rats

**DOI:** 10.1515/biol-2022-0885

**Published:** 2024-06-18

**Authors:** Hong-jin Wang, Xiao-xia Tian, Ke-wei Zhang, Jian-zhang Niu, Shi-juan Mei, Li-zhuang Hao, Yi Li

**Affiliations:** Department of Burns and Plastic Surgery, Affiliated Hospital of Qinghai University, Xining, 810012, China; Key Laboratory of Plateau Grazing Animal Nutrition and Feed Science of Qinghai Province, Academy of Animal Science and Veterinary Medicine, Qinghai University, Xining, 810016, China; Department of Oncology, Affiliated Hospital of Qinghai University, Xining, 810010, China

**Keywords:** basal feed, biochemistry testing, diet ratio, routine blood examination

## Abstract

To evaluate the effects of varying proportions of yak meat in feed on the growth of rats and provide a theoretical basis for selecting the optimal feed proportion suitable for rats. This study was designed as a one-variable experiment. Fifty male rats were divided into five groups. The ratios of yak meat to basal feed of rats in four dietary treatment groups were 2:8, 4:6, 6:4, and 8:2, respectively, while those in the control group were only provided a basal diet. In the feeding experiment, the body weights of the rats were recorded on Day 0 and subsequently in the first, second, third, and fourth weeks, along with quantities of feed intake. The body and tail lengths, as well as the waist circumference of the rats, were measured, and blood samples were collected in the fourth week for routine blood and biochemistry investigations. The rats in the 4:6 feed group had the best body condition. They had normal body and tail lengths, smaller waist circumferences, good posture, and were in better overall health than rats in the other groups. The results indicate that the 4:6 diet was optimal for enhancing rats’ growth performance compared to the other diets.

## 1 Background

Yak meat is characterized by its high content of protein and moisture, low fat and ash content, abundant amino acids, and rich mineral profile. It is also juicy, rich in flavor compounds [1], and safe for consumption as it is free from contamination [2]. Past studies on yak meat have yielded some notable results on its nutritive value, highlighting its abundance in vitamins and mineral elements, notably iron (Fe), which confers significant health benefits [2]. Yak meat is rich in amino acids, including essential amino acids, that are recognized worldwide as ideal protein components. All these qualities indicate that yak beef is very beneficial to human health [3–5].

In their study on mice, Cao et al. found that, when the dietary protein and energy intake were the same, a beef diet could control the body weight, improve grip strength, prolong swimming endurance, increase glycogen levels, and strengthen the antioxidant capacity of mice [6]. In another mouse diet experiment, mice fed yak placenta powder showed significantly improved tolerance, oxygen endurance, and swimming duration, along with increased weight of their immune and reproductive organs [7]. Zhao and others found that supplementation with yak bone powder could increase bone density with a higher calcium apparent absorption rate [8]. Yak active protein was shown to inhibit cell damage caused by radiation and was beneficial in gradually restoring peripheral blood, enhancing the body’s antioxidant enzyme activity, reducing lipid peroxide accumulation in the body, protecting bone marrow DNA and immune organs, and improving the body’s immunity. It effectively regulated the secretion and expression of cytokines, inhibited cell apoptosis, and demonstrated good radioprotective properties [9].

The optimal proportion of yak meat in feed that is conducive to optimal growth in rats remains undetermined. Therefore, in this article, a preliminary experiment using feeds with different yak meat proportions to determine which is most suitable for rat growth has been described. The results of this study provide a scientific basis for subsequent research efforts, especially in understanding wound healing mechanisms in scalded rats.

## 2 Materials and methods

### 2.1 Animals and materials

Experimental Sprague-Dawley (SD) rats (150 ± 10 g body weight), feed, and bedding were procured from Xi’an Koau Biotechnology Co., Ltd (Shaanxi, China; permit no.: SCXK [Shaanxi] 2018-001). Yak meat was sourced from Datong Fengjuhe Agriculture and Animal Husbandry Technology Co., Ltd.


**Ethical approval:** The research related to animal use has been complied with all the relevant national regulations and institutional policies for the care and use of animals, and has been approved by the Ethics Committee of Affiliated Hospital of Qinghai University (No. P-SL-2020089).

### 2.2 Experimental design

Fifty SD male rats with similar body weights were randomly divided into five groups, each consisting of ten rats. The experiment adopted a one-variable four-level design, consisting of a control group (that received basal feed only), a 2:8 group (fed with 20% yak meat), a 4:6 group (fed with 40% yak meat), a 6:4 group (fed with 60% yak meat), and an 8:2 group (fed with 80% yak meat). All rats were housed in a standard animal breeding facility for 28 days.

### 2.3 Feed preparation

Yak tenderloin and basal feed were ground separately and subsequently mixed at ratios of 0:10, 2:8, 4:6, 6:4, and 8:2. The resulting mixtures were mechanically pelletized, dried at 65°C, and stored at −20°C until required for further use.

### 2.4 Determination of conventional nutrients in test feeds

The moisture content of the test feeds was determined following the guidelines outlined in protocol GB/T 6435-2014 titled “*Determination of Moisture Feedstuffs*.” Ash content was assessed using the guidelines of protocol GB/T 6438-2007 titled “*Animal Feeding Stuffs: Determination of Crude Ash.*” The content of crude protein (CP) was determined as per the protocol detailed in GB/T 6432-2018 titled “*Determination of Crude Protein in Feeds: the Kjeldahl Method.*” Ether extracts (EE) were prepared using the guidelines detailed in protocol GB/T 6433-1994 titled “*Method for the Determination of Crude Fat in Feedstuffs*.” The neutral detergent fiber content was determined using the protocol GB/T 20806-2006, “*Determination of Neutral Detergent Fiber in Feedstuffs.*” Acid detergent fiber content was determined using protocol guidelines as per GB/T 20805-2006 titled “*Determination of Acid Detergent Fiber in Feedstuffs*.” Calcium content was determined according to the methodology detailed in protocol GB/T 6436-2002 titled “*Determination of Calcium in Feeds*.” Phosphorus content was determined using GB/T 6437 2002 “*Determination of Total Phosphorus in Feeds: the Spectrophotometric Method*” protocol guidelines. The energy calorie was determined as previously described [10].

The basal feed composition and nutrient levels are shown in Table 1. The specific feed composition and corresponding nutrient levels are shown in Table 2.

**Table 1:** Basic feed composition and nutrient level

Main raw materials	Nutrient	Measured nutrient level (%)
Protein	Moisture (g) ≤ 100	Moisture 7.96
Fat	CP (g) ≥ 180	Ash 8.55
Fiber	EE (g) ≥ 40	CP 18.53
Carbohydrate	Crude fiber (g) ≤ 50	Fat 2.30
Vitamins	Crude ash ≤ 80	NDF 52.35
Mineral substances	Calcium (g) 10–18	ADF 37.21
		Calcium (Ga) 1.09
	Total phosphorus (g) 6–12	Phosphorus (P) 0.97
	Ratio of calcium to total phosphorus is 1.2:1–1.7:1	Total energy 9.69
	Lysine (g) ≥ 8.2	
	Methionine + cystine (g) ≥ 5.3	

**Table 2:** Feed composition and nutrient levels

Group	2:8	4:6	6:4	8:2
Raw materials	20% yak meat	40% yak meat	60% yak meat	80% yak meat
	80% basal feed	60% basal feed	40% basal feed	20% basal feed
Moisture (%)	4.85	4.41	7.4	10.02
Ash (%)	8.91	8.99	8.76	8.57
CP (%)	22.31	24.84	28.0037	35.69
EE (%)	3.96	15.11	9.28	12.18
Neutral detergent fiber (%)	42.17	36.60	55.22	55.40
Acid detergent fiber (%)	46.43	55.09	46	40.68
Ca (%)	0.68	1.27	0.59	1.18
P (%)	1.45	0.86	0.76	0.69
Energy (MJ/kg)	10.48	11.73	12.14	13.17

### 2.5 Feeding and management

Each group of ten rats used in this study was assigned a cage with natural ventilation for feeding, and the bedding was changed regularly to maintain dry conditions. The rats were kept at a controlled temperature of 23 ± 2℃ and humidity was maintained at 40 ± 5%. They were provided *ad libitum* access to feed and water throughout the experiment.

### 2.6 Test method

After daily feeding, the initial and final body weights (weighed at noon on the following day) were recorded to calculate the SD rats’ daily feed intake and average daily weight gain. Their average daily gain was calculated as (final body weight − initial body weight)/7. Their daily feed intake was calculated as feed intake on the previous day minus the surplus on the current day.

Body length was measured from the nasal tip to the tail root. Tail length was measured from the tail’s root to the tip. The waist circumference was measured around the rat’s waist and abdomen.

Samples were collected on the 28th day of the experiment. After a 12 h fasting period, the rats were anesthetized using 10% chloral hydrate. Subsequently, they were weighed, and their body and tail lengths, as well as their waist circumference, were recorded. Blood samples were collected from their abdominal aorta using ethylenediaminetetraacetic acid (EDTA) and coagulation-promoting tubes. The EDTA tubes were stored at −20°C for routine blood examinations. The coagulation-promoting tubes were centrifuged to collect the serum, which was stored in cryogenic vials at −80°C until required. The EDTA tubes (blood samples) and cryogenic vials (serum samples) were sent to the Qinghai University Affiliated Hospital for routine blood examination and biochemistry testing.

### 2.7 Statistical analyses

Excel 2010 software was used to manage the original data, and SPSS 20.0 software was used to perform significance testing using the least significant difference (LSD) method to make multiple comparisons. Quantitative data conforming to a normal distribution were represented as mean ± standard deviation. A one-way analysis of variance was used for comparisons between groups. After the homogeneity test, the LSD test was used for comparison within groups in pairs. A *P* value of <0.05 was considered statistically significant. The experimental results were visually represented as broken line graphs and histograms created with Origin 8 software.

## 3 Results

### 3.1 Feed intake and body condition of rats fed different yak meat proportions

**Figure 1:**
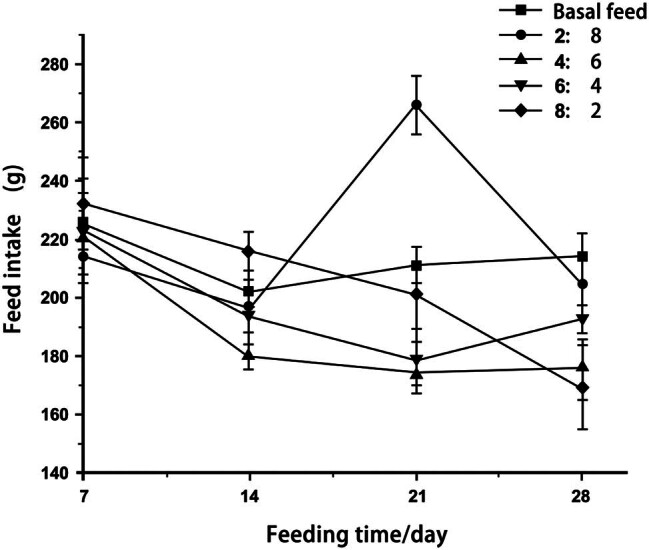
Feed intake of rats in different proportions.

Feed intake in the first week was greater than in the fourth week for all groups (Figure 1), indicating that feed intake decreased over time. Rats in the basal feed and 6:4 feed groups showed increased intake in the fourth week compared to the third week, but the intake levels were still lower than those in the first week. The intake levels of rats in the 8:2 and 4:6 feed groups decreased relatively steadily over time.

**Figure 2:**
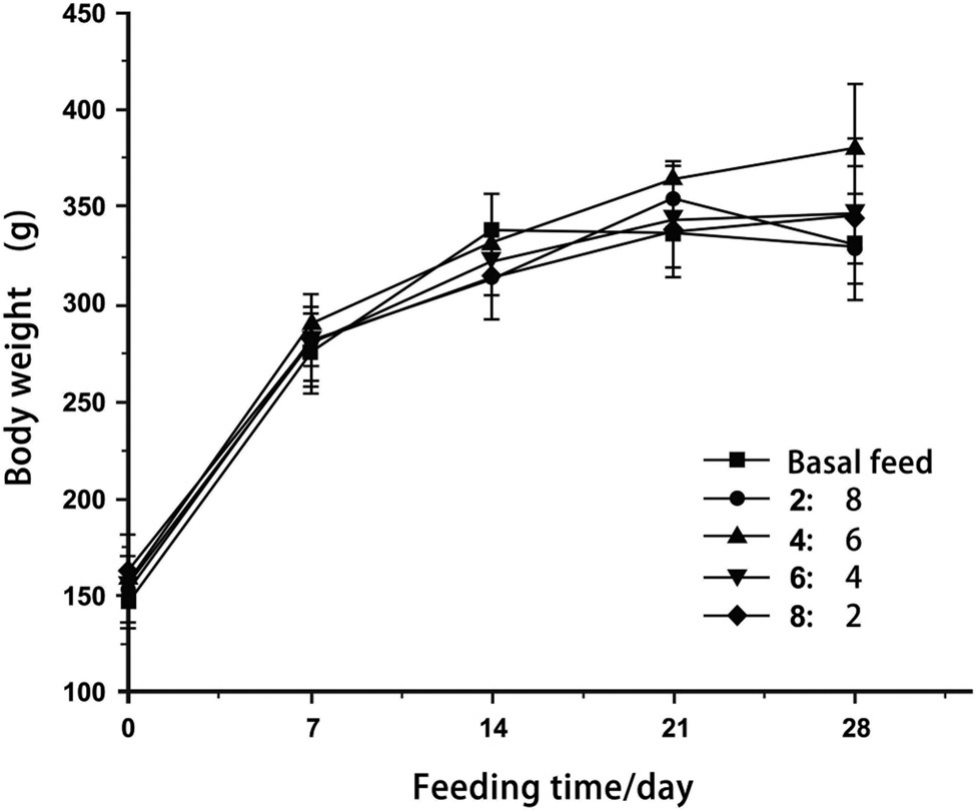
Weight of rats fed with different proportions.

The body weight of rats across all groups increased rapidly in the first week, while growth decelerated in the second and third weeks (Figure 2). Rats in the basal and 2:8 feed groups showed a decrease in their body weight in the fourth week as compared to the third week. Body weight changes in the 8:2 feed group tended to be gradual. In the 6:4 and 4:6 feed groups, the rats’ body weight increased from the third week on, although the increase in the 4:6 feed group was more significant.

**Figure 3:**
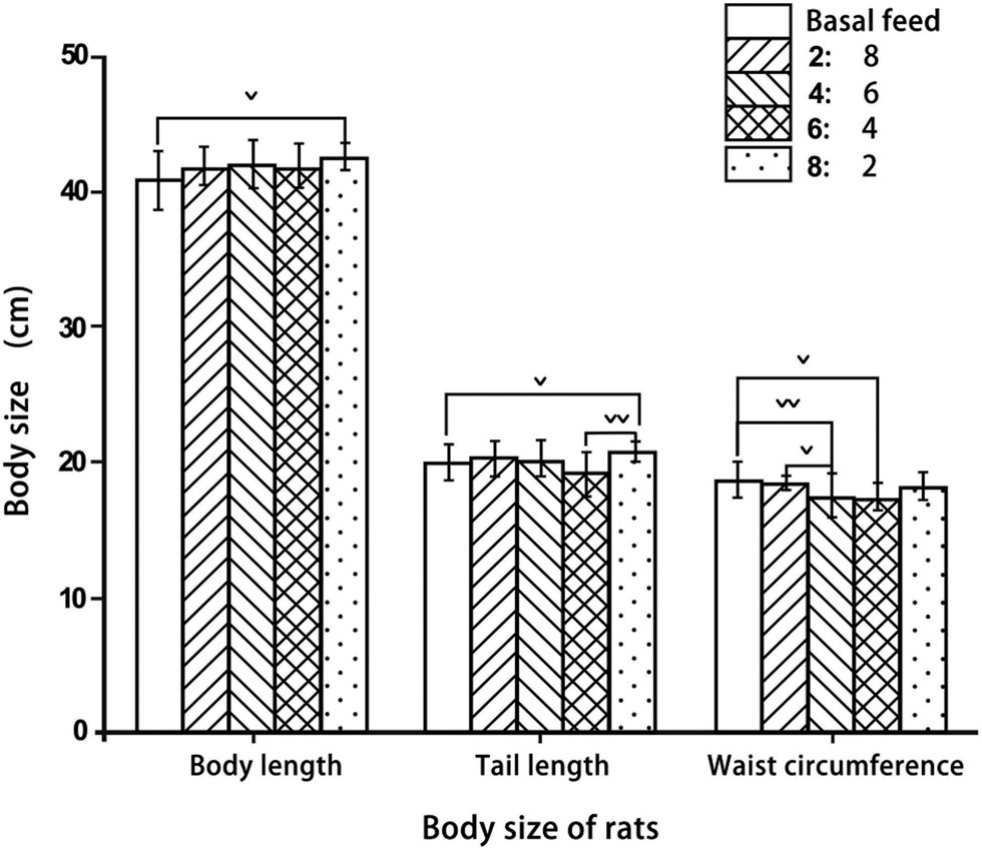
Body size of rats fed with different proportions.

Rats in the basal feed group had the shortest body lengths, and those in the 8:2 feed group had the longest body lengths (Figure 3). However, there were no significant differences in body length among the other groups.

The tail lengths of rats were significantly shorter in the 2:8 feed group than in the 8:2 feed group. They were also much shorter in the 6:4 feed group than in the 8:2 feed group.

Waist circumferences were significantly greater in the basal feed group than in the 4:6 and 6:4 feed groups. Rats in the 2:8 feed group also had significantly greater waist circumference compared to those in the 4:6 feed group.

### 3.2 Hematological indices of rats fed with different yak meat proportions

There were differences in hematological indices observed in rats fed with proportions of different yak meat in their feed (Table 3). White blood cells, lymphocytes, hemoglobin, red blood cells, and cholesterol (CHOL2) did not differ significantly among groups (*P* > 0.05). However, lymphocyte and hemoglobin levels were higher than the normal range among rats in the 2:8, 4:6, 6:4, and 8:2 groups. Red blood cell counts were above the normal range in all groups, and CHOL2 levels were lower than the normal range among rats in the 2:8 and 8:2 groups.

**Table 3:** Blood indexes of rats in different proportions of diet

Basal feed		2:8	4:6	6:4	8:2	SEM	*P*	Range of normal value
White blood cells (10^9^/L)	5.13	7.15	8.55	9.29	6.64	0.62	0.77	5.22–12.94 [6]
Lymphocytes (10^9^/L)	3.89	5.29	7.19	7.29	5.16	0.54	0.72	3.69–4.57 [7]
Hemoglobins (g/L)	157.25	163.4	163.6	169.5	176	3.18	0.098	118.25–161.09 [8]
Red blood cells (10^12^/L)	8.07	8.52	8.26	8.56	9.2	0.16	0.086	6.12–7.159 [6]
Platelets (10^9^/L)	668.24^b^	968^ab^	1021.6^ab^	1152^a^	945^ab^	53.51	0.033	862.21–1415.82 [6]
ALT (u/L)	63^a^	42^b^	46.2^b^	39.8^b^	44^b^	2.03	0.02	19.82–51.95 [8]
TP (g/L)	60.06^a^	52.9^b^	54.08^b^	51.96^b^	53.82^b^	0.73	0.038	47.4–58.7 [9]
ALB (g/L)	35.1^a^	30.88^b^	31.84^b^	30.82^b^	31.38^b^	0.41	0.044	22.16–42.53 [8]
TRIG (mmol/L)	0.8^a^	0.29^b^	0.31^b^	0.36^b^	0.37^b^	0.49	0.000	0.63–0.719 [10]
CHOL2 (mmol/L)	1.15	0.86	1.17	1.17	0.96	0.47	0.641	1.07–2.09 [9]

Note: Values within a row with different lowercase letters as superscripts differ significantly.

Rats in the 6:4 feed group had significantly increased platelet levels than those in the basal feed group (*P* < 0.05); however, these levels did not differ significantly among the other groups. Platelet levels were below the normal range among rats in the basal feed group.

Alanine aminotransferase (ALT), total protein (TP), albumin (ALB), and triglyceride (TRIG) levels were significantly higher among rats in the basal feed group than in the other groups (*P* < 0.05). ALT and TP levels exceeded the normal range in the basal feed group. TRIG levels were above the normal range among rats in the basal feed group but below the normal range in the other groups.

## 4 Discussion

### 4.1 Effects of feed with different yak meat proportions on the feed intake and body weight of rats

Feed intake and body weights differed between rats who were fed different proportions of yak meat in their feed. The feed intake of rats in all groups decreased over time. Prolonged access to high-fat (HF), high-protein (HP), or high-carbohydrate diets can influence short-term regulation of food intake. An HP diet may increase sensitivity to intestinal satiety signals, whereas an HF diet may decrease sensitivity [11–14].

Liu et al. showed that the feed intake of rats fed on HF and HP diets increased rapidly, followed by a gradual decline, while rats that were fed a conventional diet showed a slow increase in feed intake. Our findings are consistent with the results of this study. These feeding patterns might indicate satiety in rats fed HF and HP diets, and the feed intake could be increased by adjusting their dietary composition [15].

The body weight of rats fed HF and HP diets increased faster initially than that of rats fed a conventional diet, followed by a gradual decrease. These results of the current study were consistent with those of an earlier investigation [15], possibly reflecting increased lipoprotein expression in the hypothalamus induced by the HF and HP diets, which could suppress the rats’ appetites and further affect their body weights.

In their study, Chen et al. [16] found that rats fed a long-term HP and low-carbohydrate diet lost weight, and this conclusion is similar to the body weight gains that were observed in our study in the 8:2, 6:4, and 4:6 feed groups in the fourth week. Gain in body weight decreased with increasing protein content over time. If the experimental duration was extended, rats that were fed the HP diet would eventually lose weight.

Rats fed a 28-day HP diet showed body weight loss and reduced feed intake, as reported by Liu et al. [17]. This finding supports the results of our study, potentially highlighting the increased energy required for meat digestion. Consequently, as the ratio of yak meat to basal feed increased, the demand for energy required for digestion and absorption also increased, contributing to body weight loss. Therefore, the decline in body weight observed in the 2:8 feed group compared to the basal fed group in the fourth week may be attributed to decreased feed intake since the provided nutrients failed to meet their daily requirements.

### 4.2 Effects of different yak meat proportions on the hematological indices of rats

There were significant differences in the hematological indices of rats that were fed different proportions of yak meat. White blood cells have protective functions depending on their physiological characteristics, and these include leukopedesis, chemotaxis, phagocytosis, and secretion [18]. White blood cell levels were significantly higher among rats in the 4:6 and 6:4 feed groups than those in other groups, yet they remained within the normal reference range. Lymphocytes, mainly involved in specific immunity, play a vital role in the body’s immune response [19]. Lymphocyte levels were higher in the 4:6 and 6:4 feed groups than in other groups, indicating that rats in these two groups had better immune function compared to the other groups.

Hemoglobin is crucial for the transport of oxygen and carbon dioxide. In this study, the hemoglobin levels of rats were above the normal range in all groups except the basal feed group, and the levels increased with the proportion of yak meat in the feed. This trend may be attributed to yaks’ adaptation to living in the hypoxic environment of the Qinghai-Tibetan plateau, making their meat rich in hemoglobin and subsequently increasing hemoglobin levels in rats that consumed this meat. Red blood cells, which protect hemoglobin and ensure it works consistently, also have an immune function. We found that the red blood cell counts exceeded the normal range in all groups, likely influenced by the high-altitude living conditions in the Qinghai region.

Platelets are essential for maintaining vascular endothelium integrity and are involved in the physiological hemostasis and blood coagulation processes. Increased platelet levels can be induced by factors such as exercise, feeding, and hypoxia. In our study, rats in the 2:8, 4:6, and 6:4 feed groups had significantly higher platelet levels than those in the other two groups, while platelet levels were lower than the normal range in the basal feed group. The proportions of yak meat in the feed appeared to play a pivotal role in the observed differences in platelet levels among rats fed in cages at the same altitude.

Additionally, based on the results, rats in the 4:6 feed group showed relatively good body growth and overall health conditions, characterized by lower feed intakes, steadier changes in parameters, normal body and tail lengths, smaller waist circumferences, and good posture compared to rats in the other groups. Therefore, it can be concluded that the 4:6 feed formula is optimal for achieving good rat growth performance.

Serum TP contains ALB, the reservoir for histones, which aids in repairing damaged tissues and maintaining a stable plasma colloid osmotic pressure [20]. A high TP content indicates a good ability for protein digestion in rats. Since rats in the basal feed group had the highest TP content, the protein content in the basal feed was possibly lower than that in other diets with the same basic conditions.

Serum ALT activity serves as an indicator of liver function and can be considered a marker for *in vivo* nutrient metabolism and stress response. Increased ALT activity indicates impaired liver function, characterized by the release of large amounts of intracellular enzymes into the blood, significantly increasing enzymatic activity in serum. In this experiment, ALT levels in the basal feed group exceeded the normal range, indicating impaired liver function in the rats in this group.

TRIGs are synthesized in the liver or small intestine and serve as the main component of very low-density lipoprotein. The highest TRIG blood levels were found in the basal feed group.

CHOL2 plays a crucial role in animals, forming part of the cell membrane and serving as the raw material for synthesizing bile acid, vitamin D3, and steroid hormones [21]. A previous study showed that total hepatic CHOL2 levels in chicken, pork, and beef were lower than those in fish [22]. In our study, rats in the 4:6 and 6:4 feed groups had the highest CHOL2 levels, suggesting that their diets had relatively high nutrient levels.

To sum up, we reasonably speculate that yak meat also has beneficial effects on human growth and development, as well as wound healing. Regular consumption of yak meat can supplement essential nutrients required by the human body, promote wound healing, and actively aid in the treatment of inflammatory diseases, making it a valuable and beneficial food in a healthy diet.

## 5 Conclusion

In conclusion, our results in this study were that the feed intake of rats decreased as the proportion of yak meat in their feed increased, consequently slowing their body weight gain. Rats in the 4:6 feed group showed a steady increase in feed intake and body weight. White blood cell, lymphocyte, platelet, and CHOL2 levels were higher among rats in the 4:6 and 6:4 feed groups than in other groups, indicating better blood nourishment in rats in these groups. Overall, feed containing 40% yak meat offered the greatest benefits for rat growth.
